# Synthesis and Characterization of a Biomimetic Formulation of Clofazimine Hydrochloride Microcrystals for Parenteral Administration

**DOI:** 10.3390/pharmaceutics10040238

**Published:** 2018-11-17

**Authors:** Mikhail D. Murashov, Jennifer Diaz-Espinosa, Vernon LaLone, Joel W. Y. Tan, Raluca Laza, Xueding Wang, Kathleen A. Stringer, Gus R. Rosania

**Affiliations:** 1 Department of Pharmaceutical Sciences, University of Michigan, Ann Arbor, MI 48109, USA; jendiaz@umich.edu (J.D.-E.); laloneve@umich.edu (V.L.); rmlaza@umich.edu (R.L.); 2 Department of Biomedical Engineering, University of Michigan, Ann Arbor, MI 48109, USA; tanjoel@umich.edu (J.W.Y.T.); xdwang@umich.edu (X.W.); 3 Department of Radiology, University of Michigan, Ann Arbor, MI 48109, USA; 4 Department of Clinical Pharmacy, University of Michigan, Ann Arbor, MI, 48109, USA; stringek@umich.edu

**Keywords:** crystal-like drug inclusion (CLDI), micronized crystals, jet milling/micronization, drug repurposing, CFZ-induced skin pigmentation, adverse drug reaction (ADR), macrophages

## Abstract

Clofazimine (CFZ) is a broad spectrum antimycobacterial agent recommended by the World Health Organization as a first line treatment for leprosy and second line treatment for multidrug resistant tuberculosis. Oral administration of CFZ leads to a red skin pigmentation side effect. Since CFZ is a weakly basic, red phenazine dye, the skin pigmentation side effect results from lipophilic partitioning of the circulating, free base (neutral) form of CFZ into the skin. Here, we developed a stable and biocompatible formulation of CFZ-HCl microcrystals that mimics the predominant form of the drug that bioaccumulates in macrophages, following long term oral CFZ administration. In mice, intravenous injection of these biomimetic CFZ-HCl microcrystals led to visible drug accumulation in macrophages of the reticuloendothelial system with minimal skin accumulation or pigmentation. In fact, no skin pigmentation was observed when the total amount of CFZ-HCl administered was equivalent to the total oral dose leading to maximal skin pigmentation. Thus, parenteral (injected or inhaled) biomimetic formulations of CFZ-HCl could be instrumental to avoid the pigmentation side effect of oral CFZ therapy.

## 1. Introduction

Clofazimine (CFZ) is a weakly basic, red-pigmented, FDA-approved, phenazine antibiotic that is included in the World Health Organization’s (WHO) List of Essential Medications as part of the antibiotic cocktail used for the standard treatment for leprosy [1,2,3,4,5]. It has been in clinical use since the 1960s and has contributed to the cure of more than 16 million people worldwide [1,2,3,4,5]. Due to its potent activity against *Mycobacterium tuberculosis*, the WHO now recommends CFZ as a second line agent against multi-drug resistant tuberculosis (MDR-TB) [3,6,7,8,9,10]. In humans, CFZ exhibits atypical pharmacokinetic properties [11,12]. In mice, following long-term (i.e., weeks) oral administration, solid drug precipitates accumulate in tissue macrophages, forming red crystal-like drug inclusions (CLDIs) that are most abundant in the liver and spleen [13,14,15]. This phenomenon has also been reported in humans treated with CFZ [11,12]. Interestingly, these biocrystals display robust stability inside macrophages and remain in the body long after discontinuation of treatment (>8 weeks) [13]. The preliminary analysis of isolated CLDIs from spleen and liver of CFZ treated mice revealed that CLDIs contained a hydrochloride salt form of the drug, which is stabilized by the acidic and high chloride concentrations present in macrophage lysosomes, while the orally-administered form of the drug is the free base [16].

In terms of its toxicological properties, the major side effect of orally administered CFZ is a strong red skin pigmentation, observed in more than 94% of patients [17,18]. CFZ-induced skin pigmentation is attributed to the circulating, soluble free base form of CFZ that partitions into the subcutaneous fat layer of the skin rather than CLDI formation and accumulation [19]. Even though prolonged CFZ treatment is associated with massive drug biocrystal accumulation within resident tissue macrophages, there are no obvious toxicological manifestations from these biocrystals. Instead, CLDIs are biocompatible, stable, long-lived, and relatively non-toxic [3,20]. Phagocytosed CLDIs modulate innate immune signaling by dampening pro-inflammatory and enhancing anti-inflammatory pathways via inhibition of tumor necrosis factor alpha (TNFα) and enhancement of interleukin 1 receptor antagonist (IL-1RA) secretion [3,20]. Specifically, CFZ inhibited carrageenan- and lipopolysaccharide-induced inflammation in the footpads and lungs, respectively, in 8-week-CFZ-treated mice, in an IL-1RA-dependent manner [3].

Since the skin pigmentation side effect is likely due to the free base form of the drug that partitions from the blood to the skin following oral administration, we decided to test whether parenteral administration of CFZ-HCl microcrystals could be used to avoid the drug’s skin pigmentation side effect. We hypothesized that the injected, micronized CFZ-HCl particles would be ingested by macrophages and stabilized by the phagolysosomal microenvironment. In order to test this hypothesis, we developed and tested CFZ-HCl particles side-by-side with CLDIs and other solid forms of CFZ, in terms of their physicochemical characteristics. Furthermore, biological experiments were conducted to test the performance of biomimetic CFZ-HCl formulations following phagocytosis by macrophages in vitro*,* and in vivo after administering the drug via intravenous (IV) tail-vein bolus injection. The results indicate that a biomimetic formulation of CFZ-HCl can be effectively synthesized and delivered parenterally to circumvent the CFZ-induced skin pigmentation side effect.

## 2. Materials and Methods

### 2.1. Synthesis of Different Clofazimine (CFZ) Solids

Different CFZ salt crystals were synthesized by adding 1 M of ammonium salts in MiliQ water to 2 mM CFZ (C8895, Sigma-Aldrich, St. Louis, MO, USA) in methanol in a 1:1 ratio (*v*/*v*). Ammonium bromide, carbonate, citrate dibasic, nitrate, phosphate dibasic, and sulfate were all purchased from Sigma-Aldrich (213349; 207861; 25102; 221244; 215996; A4418 (respectively); Sigma-Aldrich, St. Louis, MO, USA). Ammonium acetate and chloride were purchased from Fisher Scientific (A639; A661 (respectively); Fisher Scientific, Fair Lawn, NJ, USA). Ammonium iodide was purchased from Acros Organics (448071000, Acros Organics, Morris Plains, NJ, USA).

### 2.2. Isolation of Crystal-Like Drug Inclusions (CLDIs)

CLDI purification and isolation were performed as previously described with minor modifications [13,14,21]. In brief, liver and spleen from mice that were fed CFZ for 8 weeks from previous experiments were homogenized by cutting the organs into smaller pieces and utilizing mechanical homogenizer (Pro200; Pro Scientific, Oxford, CT, USA). Liver and spleen homogenates were filtered through a 100 μm strainer, and the filtrate was collected in a 50 mL centrifuge tube and diluted up to 50 mL with 1× Phosphate Buffered Saline (PBS) (10010023, Gibco, Life Technologies, Carlsbad, CA, USA). The filtrate was then centrifuged at 250× *g* for 6 min. The supernatant was discarded, and fresh PBS was added up to 50 mL. This step was repeated twice, and after the final washing step, the red-CLDI containing pellet was resuspended in 2–5 mL of 10% sucrose. From the pellet, CLDIs were further purified using a three-layer discontinuous gradient (50%, 30%, and 10% sucrose in PBS) centrifugation method (3200× *g*, 60 min). After centrifugation, the top layers were discarded, and the CLDIs pellet was resuspended in PBS and snap frozen in liquid nitrogen in preparation for future analysis.

### 2.3. Bulk Synthesis of CFZ-HCl Salt Crystals

CFZ free base (10 g; Sigma-Aldrich, St. Louis, MO, USA) was placed in 2 L of 1 M HCl in an Erlenmeyer flask with a magnetic stir bar. After the addition of CFZ, the reaction was sealed with parafilm and stirred in the dark for 72 h. After 72 h, the stirring was turned off and CFZ salt crystals were allowed to precipitate. Following crystal precipitation, the HCl reactant was decanted and discarded. The residual acid containing the CFZ salt crystals was centrifuged (2000× *g*, 10 min, 4 °C), the supernatant was discarded, and the pellet was washed thrice with declining concentrations of HCl (100 mM, 10 mM, and 1 mM) to reduce the amount of chloride present in the solution. In between washing steps, samples were centrifuged (2000× *g*, 10 min, 4 °C). After the last wash, crystals were resuspended in MiliQ water and immediately snap frozen in liquid nitrogen and then freeze-dried.

### 2.4. Micronization (Jet Milling) and Sterilization

A SepSol, Sturtevant Inc. (Separation Solutions; SepSol Process Solutions, Kalamazoo, MI, USA) air jet mill was used to micronize the bulk synthesized CFZ-HCl crystals and CFZ free base crystals (control) to a particle size distribution within the desired range (0.5–5 µm). Following the company’s specifications, the air jet mill was set at 50 psi grind pressure and 100 psi feed pressure. Filtered, dried, compressed air was used as the air source for the mill. Altogether, four batches of CFZ-HCl microcrystals (net weight ~40 g) and three batches of CFZ free base microcrystals (net weight ~20 g) were produced with ~80–90% yield from milling. Both types of micronized CFZ crystals were stored at −20 °C until the time of experimentation. Before each experiment, CFZ microcrystals were sterilized by dry heat at 170 °C for 1.5 h, using a bench top vacuum oven at 5 psi (Model 5831; National Appliance Co., Portland, OR, USA).

### 2.5. Particle Size Determination

The CFZ microcrystals particle size after synthesis and after micronization were analyzed using Zeta-Sizer (Malvern Instruments, Nano-ZS90, Malvern, UK) and brightfield microscopy using Nikon Eclipse Ti inverted microscope (Nikon Instruments, Melville, NY, USA).

### 2.6. Proton Nuclear Magnetic Resonance Spectroscopy Analysis

Proton (^1^H) nuclear magnetic resonance (NMR) spectroscopy was performed as previously described [16]. Briefly, one-dimensional ^1^H-NMR spectra of micronized CFZ-HCl crystals at different stages of manufacturing were acquired using an 11.74 T (500 MHz) NMR spectrometer with a VNMRS console and a 7510-AS autosampler system operated by host software VNMRJ 3.2 and equipped with a 5 mm Agilent One NMR probe with Z-axis gradients. The samples were dissolved in dimethyl sulfoxide-D6 (D, 99.9%) (DMSO-*d*_6_; Cambridge Isotope Laboratories, Inc., Andover, MA, USA). The NMR data were acquired at room temperature and processed using MestreNova 9.0 software (MestreLab, Santiago de Compostela, Spain).

### 2.7. Powder X-ray Diffraction Analysis

Powder X-ray diffraction (pXRD) spectra of micronized CFZ-HCl crystals at different stages of manufacturing were taken by Rigaku Miniflex X-ray diffractometer (Rigaku-USA Inc., Danvers, MA, USA) using Cu Kα radiation, a tube voltage of 30 kV, and a tube current of 15 mA. Measurements were taken from 5° to 40° at a continuous scan rate of 2.5°/min.

### 2.8. Raman Microscopy

Raman spectra were acquired with a WiTec alpha300R confocal Raman microscope (WiTec, Ulm, Germany) equipped with a 532 nm solid-state sapphire excitation laser and charge coupled device (CCD) detector. For single point spectra, the laser was focused on the sample acquiring each Raman spectrum with an integration time of 75 s, using Zeiss EC EPIPLAN 50X objective (N.A. = 0.75). Alternatively, for large area scans, the laser rastered across a 100 µm by 100 µm area of the sample with a step size of 10 µm and an integration time of 2 s per pixel, using Zeiss 10 X objective. To measure quality and stability of individual CFZ microcrystals, single point spectra were taken from individual particles for micronized CFZ-HCl, micronized CFZ-HCl in diluent, and micronized CFZ free base that were dispersed on mica chips. Cosmic rays were removed from all spectra using the WiTec Project FOUR software. A MATLAB^®^ processing algorithm developed in-house [22] was used to baseline-subtract, normalize, and overlay spectra to qualitatively identify the collected micronized sample spectra by comparing them to both CFZ-HCl and CFZ free base reference spectra.

### 2.9. Brightfield and Fluorescence Microscopy

Microscopy was performed using a Nikon Eclipse Ti inverted microscope (Nikon Instruments, Melville, NY, USA) as previously described [19,23]. Briefly, brightfield images were captured using the Nikon DS-3 camera (Nikon Instruments, Melville, NY, USA), and fluorescence imaging in FITC channel (490/510 nm, green) and Cy5 channel (640/670 nm, far-red) was performed with the Photometrics CoolSnap MYO camera system (Photometrics, Tuscon, AZ, USA) under the control of Nikon NIS-Elements AR software (Nikon Instruments, Melville, NY, USA). Illumination for fluorescence imaging was provided by the X-Cite 120Q Widefield Fluorescence Microscope Excitation Light Source (Excelitas Technology, Waltham, MA, USA).

### 2.10. Assaying Stability of CFZ-HCl in Aqueous Buffers

The stability of CFZ-HCl microcrystals was evaluated in IV diluent, synthetic lysosomal buffer [19], and PBS. A small amount of CFZ-HCl crystals (<1 mg) was placed in a large volume of test media (~20 mL) and gently stirred for 24 h to make sure that the equilibrium between the microcrystals and the test medium was reached. After 24 h, the small drop of test media with microcrystals was placed on the glass microscope slide, and the stability of CFZ-HCl microscrystals was evaluated using brightfield and fluorescence microscopy (see Section 2.9).

The diluent for the IV injectable formulation was made using polysorbate 80 (59924 Sigma-Aldrich, St. Louis, MO, USA), sodium chloride (BP358, Fisher Scientific, Fair Lawn, NJ, USA), and Milli-Q water. To coat, disperse, and adjust the size of the lipophilic CFZ-HCl microcrystals, the varying concentrations of polysorbate 80 (0–0.5%) was used [24]. Sodium chloride was added to maintain isotonicity. The pH was adjusted to pH 5 using 0.01 M HCl or 0.01 M NaOH to ensure the stability of CFZ-HCl microcrystals in the formulation. For experiments, the diluent was sterilized by sterile filtration with a syringe filter (09-719A; 0.22 µm, MCE, Sterile; Fisher Scientific, Fair Lawn, NJ, USA).

The synthetic lysosomal buffer was prepared as previously reported [19]. In brief, synthetic lysosomal buffer was made by preparing 10 mM sodium acetate buffer (pH 4.5) with 10 mM cetrimonium bromide (CTAB) and 100 mM NaCl to mimic lysosomal conditions [19].

### 2.11. Cell-Based Stability Assays

The stability of CFZ-HCl microcrystals was evaluated following phagocytosis, using the RAW 264.7 murine macrophage cell line. The cell line was purchased from ATCC (Manassas, VA, USA) and maintained in Dulbecco’s modified Eagle medium (DMEM) (11995-065, Gibco, Life Technologies, Carlsbad, CA, USA) supplemented with 10% fetal bovine serum (FBS) (16000-044, Gibco, Life Technologies, Carlsbad, CA, USA) and 0.1% penicillin/streptomycin (15140-122, Gibco, Life Technologies, Carlsbad, CA, USA). Cells were maintained at 5% CO_2_ at 37 °C and passaged at 80% confluency. For the assay, the cells were seeded at 5 × 10^4^ cells/well in 6-well plates and were allowed to grow for 24 h, at which point, isolated CLDIs, CFZ-HCl microcrystals, and CFZ free base microcrystals were added at 20 µg/mL in DMEM with 5% FBS to designated drug treatment plates. The same volume of DMEM with 5% FBS without drug treatment was added to wells of negative control plates. The cells were treated for 1 h, at which point, the media was removed, and the cells were thoroughly washed twice. DMEM with 10% FBS and 0.1% penicillin/streptomycin was then added back into the wells (*t*_0_). Over the next 48 h (*t*_8h_, *t*_24h_, *t*_48h_), total cell viability was assessed using trypan blue (0.4%) exclusion (15250-061, Gibco, Life Technologies, Carlsbad, CA, USA). At every time point, brightfield and fluorescence images were taken, and the viability measurements were calculated in triplicate (# cells >100 per measurement).

### 2.12. Animal Studies

Animal care was provided by the University of Michigan’s Unit for Laboratory Animal Medicine (ULAM), and the experimental protocol was submitted to and approved by the University of Michigan’s Institutional Committee on Use and Care of Animals (PRO00007593; 5 May 2017).

In order to assess of the skin pigmentation induced by micronized CFZ-HCl crystals following injection into the systemic circulation, 14 male mice (10–19 weeks old, C57BL6, Jackson Laboratory, Bar Harbor, ME, USA) were assigned to one of 3 groups: CFZ orally fed (OF) group (*n* = 5), CFZ-HCl IV injected group (*n* = 5), and the control group (*n* = 4). The CFZ orally fed group and control group mice were fed CFZ or vehicle chow for 9 weeks, respectively, as previously reported [13,14,20]. The CFZ-HCl IV injected group received a single IV bolus tail-vein injection with the formulation of CFZ-HCl microcrystals (200 mg/kg), using a 300 μL injection volume, which translates to an approximate total dose of 6 mg of drug microcrystals or 3 weeks of orally administered CFZ (assuming 100% bioavailability). Animals were sacrificed 24 h post injection. At the time of sacrifice, mice were euthanized with CO_2_ inhalation and exsanguination.

#### 2.12.1. Toxicological Evaluation

The mice were monitored for behavioral changes and survival (e.g., lethargy, scruffy fur, squinted eyes, etc.) 24 h post-injection to evaluate the toxicity of the CFZ-HCl injections. In addition, plasma samples from the in vivo experiments were assayed for IL-1RA, IL-1β, and TNFα by enzyme linked immunosorbent assay (ELISA; Quantikine; R&D Systems, Minneapolis, MN, USA) in duplicate according to the manufacturer’s instructions. The cytokine concentrations were expressed as picograms per milliliter of plasma.

#### 2.12.2. Immunohistochemistry

Immunohistochemistry was performed using a previously published protocol with some modifications [25]. In brief, following euthanasia, the liver and spleen were removed en bloc and embedded in Tissue-Plus Optimal Cutting Temperature (OCT) compound (4585, Fisher HealthCare, Houston, TX, USA). Frozen tissue blocks were sectioned (6 µm thick) using Leica 3050S cryostat, fixed in 4% paraformaldehyde (15710, Electron Microscopy Sciences, Hatfield, PA, USA) for 10 min, and blocked with 1% bovine serum albumin (BSA) (810033, MP Biomedicals, Solon, OH, USA), 5% goat serum (Sigma-Aldrich, St. Louis, MO, USA), and 0.3 M glycine (G8898, Sigma-Aldrich, St. Louis, MO, USA) in PBS for 2 h. The samples were then incubated with primary antibodies, anti-CD68 antibody (ab53444; 1 mg/mL stock; 1:200 dilution in 1% BSA; Abcam, Cambridge, UK) or purified rat IgG2a κ isotype control antibody (400502; 0.5 mg/mL stock; 1:100 dilution in 1% BSA; Biolegend, San Diego, CA, USA), overnight at 4 °C, followed by incubation with anti-rat IgG (H + L) Alexa Fluor^®^ 488 conjugate secondary antibody (4416; 5 mg/mL stock; 1:500 dilution in 1% BSA; Cell Signaling Technology, Danvers, MA, USA) for 1 h at room temperature. Finally, the samples were incubated with Hoechst 33,342 solution (H3570; 1 µM stock; 1:10,000 dilution in PBS; Life Technologies, Carlsbad, CA, USA) for 10 min at room temperature for nuclear detection. After staining was complete, sections were mounted with a drop of ProLong^®^ Gold antifade reagent (P36930, Life Technologies, Carlsbad, CA, USA) and sealed with a cover slip. Brightfield and fluorescence images were acquired as previously described in Section 2.9.

#### 2.12.3. Imaging and Quantification of Skin Pigmentation

Imaging and quantification of mouse skin samples were performed using the previously published protocol [19]. Briefly, two single-band bandpass optical filters were used: 480 nm (D480/30x; Chroma, Bellows Falls, VT, USA) and 623 nm (FF01-623/24–25; Semrock, Brightline, Rochester, NY, USA). These filters were attached to an iPhone SE camera for image acquisition. The flash and high dynamic range options on the camera were disabled, and the camera editing filters were not applied. Quantification analysis of pigmentation was performed using ImageJ image processing software [26].

### 2.13. Statistics

Statistical analyses were performed using SPSS Statistics Software (Version 24; IBM Corp, Armonk, NY, USA). Data are expressed as the mean ± SD. A one-way analysis of variance single factor followed by either a Tukey’s honest significant difference or Games-Howell post hoc test were used to determine significant differences when applicable.

## 3. Results and Discussion

**Figure 1:**
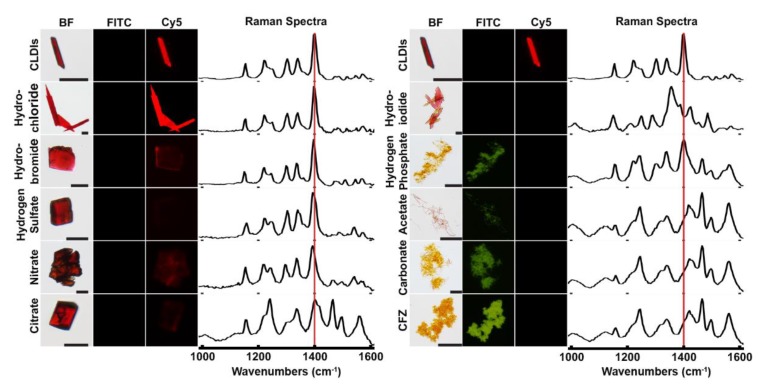
Single crystal Raman micro-spectroscopy spectra and brightfield (BF)/fluorescent (FITC, Cy5) microscopy images of various synthesized clofazimine (CFZ) salts compared to crystal-like drug inclusions (CLDIs) and CFZ free base. The red line represents the signature peak of CLDIs at 1400 cm^−1^. Scale bar = 20 µm.

First, we synthesized a focused library of CFZ salts (hydrochloride, hydrobromide, hydrogen sulfate, nitrate, citrate, hydroiodide, hydrogen phosphate, acetate, and carbonate salts) and compared their fluorescence and Raman spectral properties. For fluorescence analysis, image data were acquired with the standard FITC and Cy5 excitation/emission channels of an epifluorescence microscope; for Raman spectral data, single crystals were analyzed and compared to spectra of isolated CLDIs (Figure 1). By visual inspection, CLDIs and CFZ-HCl exhibited almost identical fluorescence and Raman spectra, sharing the CLDIs signature peak at 1400 cm^−1^ (red line). CFZ-hydrobromide, CFZ-hydrogen sulfate, and CFZ-nitrate had similar Raman spectra when compared to CLDIs and CFZ-HCl; however, the Cy5 signal was much less prominent. Furthermore, based on CLDIs signature peak, the most prominent spectral peak of CFZ-hydrobromide, CFZ-hydrogen sulfate, and CFZ-nitrate showed a small shift in wavelength. All other CFZ salt crystals displayed a completely different Raman spectrum or possessed different visual/fluorescent profile compared to CLDIs. Thus, CFZ-HCl salt crystals most closely mimicked the optical properties of CLDIs.

**Figure 2:**
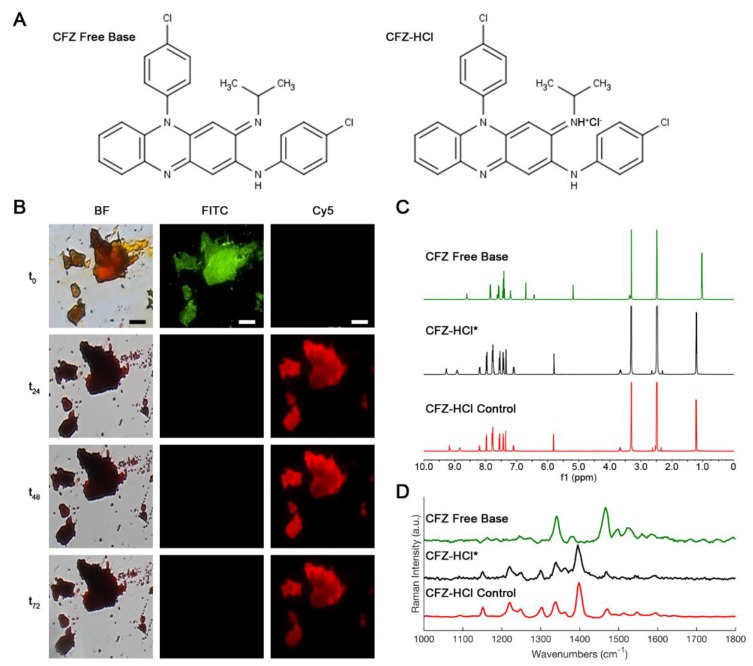
Chemical structures of CFZ free base and CFZ-HCl (**A**). Brightfield (BF)/fluorescent (FITC, Cy5) microscopy images (**B**); ^1^H-NMR (**C**), and Raman micro-spectroscopy spectra (**D**) of synthesized CFZ-HCl salt crystals via new synthesis scheme (*) compared to the established CFZ-HCl salt crystals and CFZ free base. Scale bar = 50 µm.

To scale the synthesis of CFZ-HCl to multigram and larger quantities for manufacturing of a pharmaceutical-grade formulation for clinical trials, a new synthesis scheme was developed using hydrochloric acid for transforming clofazimine free base to hydrochloride salt crystals directly, without dissolving the free base in organic solvents. For free base particles of ~100 µm diameter or less, direct conversion hydrochloride crystals were complete by 72 h, as determined using microscopy, ^1^H-NMR, and Raman (Figure 2).

The protonation state of the CFZ molecule in the different CFZ crystal forms was examined via solution ^1^H-NMR studies, conducted in DMSO-*d*_6_. ^1^H-NMR data of newly synthesized CFZ-HCl crystals (CFZ-HCl*) depicts: ^1^H NMR (400 MHz, DMSO-*d*_6_) δ 9.29 (s, 1H), 8.94 (s, 1H), 8.26–8.17 (m, 1H), 8.04–7.95 (m, 2H), 7.79 (m, 4H), 7.60–7.53 (m, 2H), 7.49–7.41 (m, 2H), 7.37 (s, 1H), 7.15–7.07 (m, 1H), 5.82 (s, 1H), 3.68 (dq, *J* = 6.5 Hz, 1H), 1.23 (d, *J* = 6.3 Hz, 6H). ^1^H-NMR data of control CFZ-HCl crystals depicts: ^1^H NMR (500 MHz, DMSO-*d*_6_) δ 9.18 (s, 1H), 8.86 (s, 1H), 8.25–8.18 (m, 1H), 8.05–7.95 (m, 2H), 7.79 (m, 4H), 7.62–7.54 (m, 2H), 7.49–7.42 (m, 2H), 7.37 (s, 1H), 7.16–7.08 (m, 1H), 5.82 (s, 1H), 3.68 (dq, *J* = 6.5 Hz, 1H), 1.23 (d, *J* = 6.4 Hz, 6H). Using our scalable synthesis method, the resulting CFZ-HCl* NMR spectra matched CFZ-HCl control spectra and was consistent with published spectra [16]. Both CFZ-HCl* and control CFZ-HCl crystals revealed the expected protonation of the tertiary amine (Figure 2A) by having an extra singlet peak at 9.29 and 9.18 ppm, respectively (minor difference in chemical shift is attributed to the difference in the pH of the samples from different synthesis schemes). Furthermore, this protonation of the tertiary amine caused the chemical shift (+δ = 0.05–0.60 ppm) for all other protons in CFZ-HCl* to the same extent as in the control CFZ-HCl spectrum with respect to the CFZ free base spectrum, which confirmed the monoprotonated characteristic of the molecule (Figure 2C) [16]. Overall, the ^1^H-NMR spectrum of CFZ-HCl* and control CFZ-HCl crystals reflected identical protonation states of the molecule.

The chemical “fingerprint” of CFZ-HCl* was further confirmed by Raman micro-spectroscopy due to the almost identical match of all of the peaks to CFZ-HCl control spectra, including the signature peak at 1400 cm^−1^. Furthermore, the aqueous solubility of CFZ-HCl* matched the aqueous solubility of CFZ-HCl control crystals of approximately 18 µM at 25 °C (data not shown) [22]. In conclusion, the final product was verified to have the same optical and fluorescence properties (Figure 2B), as well as the same chemical characteristics, according to ^1^H-NMR and Raman micro-spectroscopy, as the reference, pure CFZ-HCl salt crystals, synthesized using the established method [16,19] (Figure 2C,D).

**Figure 3:**
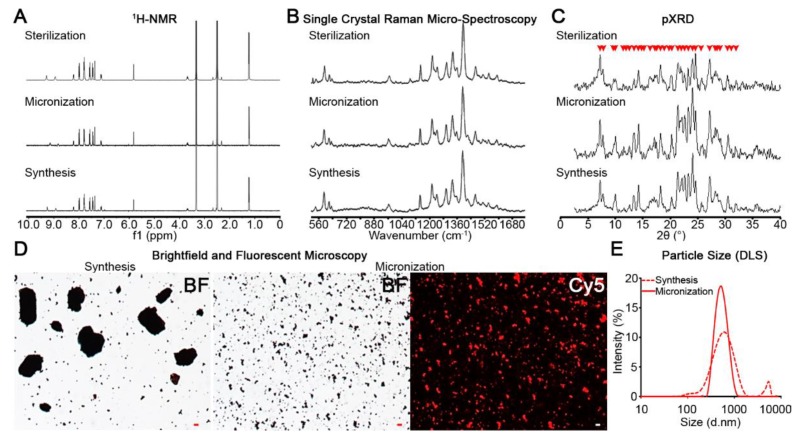
The integrity of the salt crystals was maintained throughout the manufacturing process (synthesis, micronization, and sterilization) of a biomimetic formulation of micronized CFZ-HCl salt crystals. This is evidenced by the spectra from ^1^H-NMR (**A**); single crystal Raman micro-spectroscopy (**B**); and powder X-ray diffraction (pXRD) (**C**). The red arrows in pXRD spectrum (**C**) indicate the peaks that are preserved after micronization and sterilization. The efficacy of micronization is depicted in brightfield and fluorescent images (**D**) and particle size distribution (**E**). Scale bar = 5 µm.

To formulate micronized CFZ-HCl salt crystals and characterize the extent to which they mimicked CLDIs, ^1^H-NMR, single crystal Raman micro-spectroscopy, and pXRD were used for comparative analysis. Following micronization and sterilization of bulk CFZ-HCl, chemical and structural characteristics of CFZ-HCl salt crystals remained relatively unchanged (Figure 3A–C). ^1^H-NMR spectral (Figure 3A) and single crystal Raman micro-spectroscopy peaks (Figure 3B) were preserved with almost identical match, indicating that the chemical structure of CFZ-HCl crystals was not altered by micronization or sterilization. Furthermore, pXRD spectra revealed that the majority of diffraction peaks were preserved as well, including the CFZ-HCl signature peak at 2θ = 7.2° that is absent in CFZ free base, indicating the preservation of the crystal structure (Figure 3C) [16].

Following dispersion of individual drug crystals in IV diluent buffer, Raman spectral analysis quantitatively confirmed that the CFZ-HCl particles from the micronized, sterilized powder, had the same properties (Table 1).

In IV diluent buffer, the micronized, sterilized microparticles were completely dispersed. Ultimately, the size and the dispersion of the particles can be adjusted by varying the concentration of the detergent present in the diluent (e.g., polysorbate 80); the higher the concentration, the smaller and more dispersed the particles are (data not shown). In this case, the IV injection, the maximum concentration of polysorbate 80 was used (0.5%) to make the particles as small and as dispersed as possible [24]. As a result, the microcrystals exhibited the expected Cy5 and FITC fluorescence of CFZ-HCl and were homogeneous in size distribution (Figure 3D). By utilizing dynamic light scattering analysis (Figure 3E), there was one narrow particle distribution of 547 ± 160 nm, an ideal size for parenteral formulations. In contrast, prior to micronization, the starting material exhibited 2 major wide particle size distributions of 621 ± 300 nm and 5035 ± 592 nm, respectively (Figure 3E). Thus, air jet milling produced a stable micronized product that could be readily sterilized, preserving a desirable size distribution.

**Figure 4:**
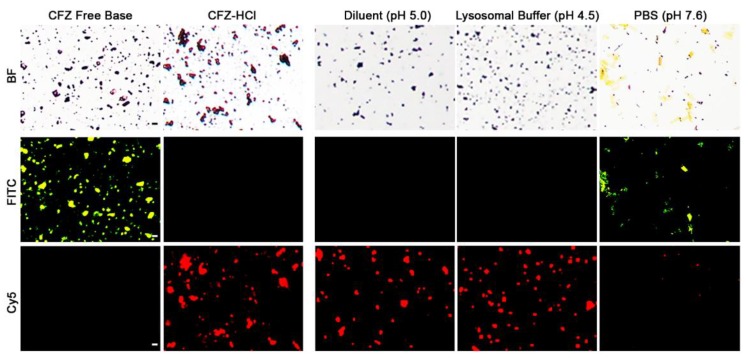
In vitro stability of the biomimetic formulation of micronized CFZ-HCl salt crystals in IV diluent, synthetic lysosomal buffer, and PBS after 24 h utilizing brightfield and fluorescent (FITC, Cy5) microscopy. Scale bar = 5 µm.

Following physical characterization, the sterilized CFZ-HCl microcrystals were tested for stability properties, by comparing fluorescence in the IV diluent, synthetic lysosomal buffer [19], and physiological saline media (PBS) (Figure 4). The micronized particles were stable in diluent (pH 5) and synthetic lysosomal buffer (pH 4.5) as evidenced by the preservation of a strong fluorescence signal in the Cy5 channel (indicative of CFZ-HCl salt) and absence of fluorescence in the FITC channel (indicative of CFZ free base) (Figure 4). In PBS (pH 7.6), the CFZ-HCl microcrystals were unstable as evidenced by the increased fluorescence in the FITC channel and decreased fluorescence in the Cy5 channel, consistent with the conversion of CFZ-HCl to CFZ free base (Figure 4). Based on these results, we inferred that the diluent would be appropriate for in vivo injection and that the particles may be stabilized by macrophages following phagocytosis.

**Figure 5:**
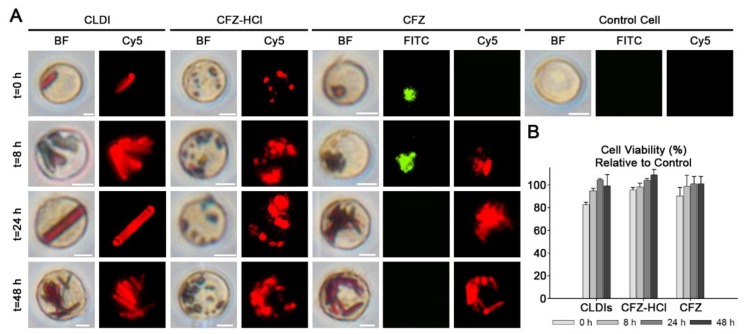
In vitro stability of the micronized CFZ-HCl salt and CFZ free base drug crystals in RAW 264.7 murine macrophages over 48 h compared to CLDIs, is depicted via brightfield and fluorescent images (FITC, Cy5) (**A**). Cell viability was not altered by 8, 24, and 48 h of treatment with CLDIs, CFZ-HCl, or CFZ free base (**B**).

To confirm this, RAW 264.7 macrophages were incubated with isolated CLDIs and micronized CFZ-HCl. The stability of the phagocytosed drug particles in live cells was microscopically evaluated following ingestion, by visual inspection (Figure 5A). RAW macrophages internalized and stabilized CLDIs as well as CFZ-HCl microcrystals, based on the strong Cy5 fluorescence signal and the absence of a FITC fluorescence signal observed in macrophages during the course of a 48-h incubation period (Figure 5A). As a control for these experiments, macrophages were incubated with CFZ free base microcrystals which fluoresce in the FITC channel, but not in the Cy5 channel. Interestingly, brightfield and fluorescent images showed that the CFZ free base gradually lost their FITC fluorescence and gained the Cy5 fluorescence, suggesting that the macrophages can transform the free base to the protonated salt form of the drug (Figure 5A).

Most importantly, incubation with CFZ-HCl was not cytotoxic. In fact, cell viability was constant (~90–100%) throughout the 48 h incubation regardless of the treatment (Figure 5B). These results were further validated by the fact that the biomimetic formulation of CFZ-HCl microcrystals were phagocytosed and stabilized by macrophages, mimicking CLDIs, which led us to conclude that these drug microcrystals would also be prone to phagocytosis and stabilization by resident tissue macrophages in vivo.

In vivo studies were performed to assess the effect of injected CFZ-HCl on the spectral reflectance properties of the skin and also to determine whether the particles accumulate in macrophages of the different organs as reported in mice fed with oral CFZ (i.e., spleen and liver) [13,14,15,19]. Since this analysis was primarily focused on assessing the extent of skin pigmentation, the IV dose (200 mg/kg), which translates to an approximate total dose of 6 mg of CFZ-HCl microcrystals (assuming 100% bioavailability) was specifically chosen to be equivalent to the amount of orally administered CFZ (total of ~6 mg, based on a bioavailability of 10mg/kg/d) [3,13,19,21] under which the maximum CFZ-induced skin pigmentation occurs when CFZ is administered orally (~3 weeks of oral administration) [19].

**Figure 6:**
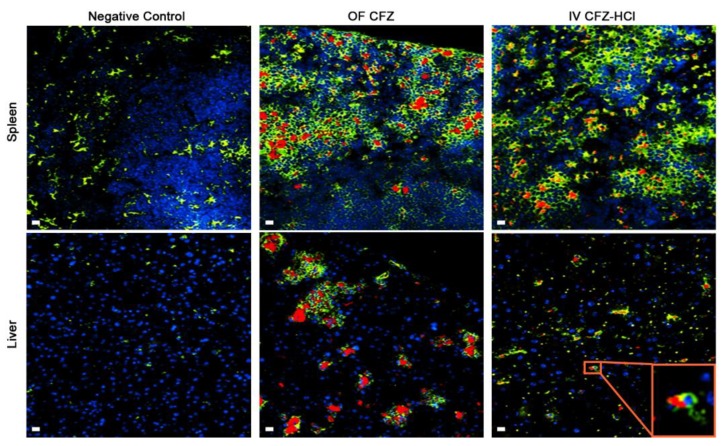
In vivo stability and phagocytosis of the biomimetic formulation of micronized CFZ-HCl salt crystals following IV injection to mice compared to 9 weeks orally fed (OF) CFZ mice. Images represent CD68 immunohistochemistry of cryo-sections of spleen and liver from OF CFZ and untreated (negative control) mice. Blue (DAPI)—nucleus; green (FITC)—CD68(+) macrophages; red (Cy5)—CLDIs/CFZ-HCl microcrystals. Scale bar = 20 µm.

All mice (*n* = 5 out of 5) that were injected with the CFZ-HCl microcrystal formulation survived the injection (100% survival) and did not show any signs of toxicity. Analyzing the distribution patterns of CFZ-HCl microcrystals in these animals, Cy5 fluorescence was observed in liver and spleen, form naturally in CFZ-fed mice (Figure 6).

**Figure 7:**
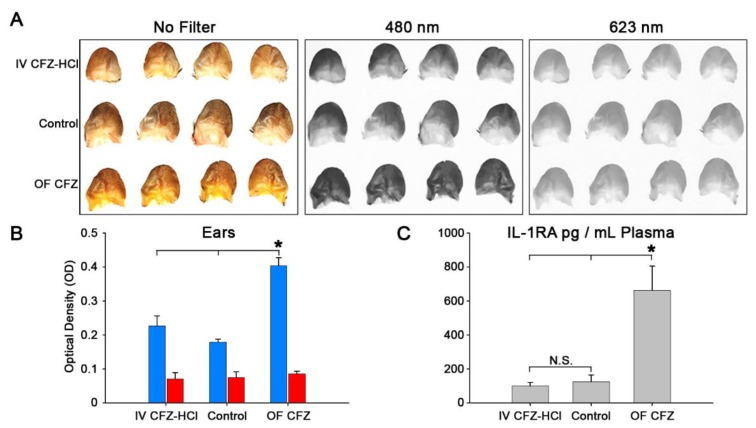
Quantitative analysis of skin pigmentation and plasma IL-1RA concentrations following IV administration of CFZ-HCl (IV CFZ-HCl) and oral administration of CFZ (9 week OF CFZ) compared to untreated mice (control). Images of mouse ears from treatment groups and the extent of their reflectance using 480 nm and 623 nm optical filters (**A**). Quantitative analysis of the pigmentation of mouse ears from CFZ treated mice compared to untreated mice (*n* = 4) via reflectance spectroscopy (blue = 480 nm; red = 623 nm filter; * *p* < 0.05, analysis of variance (ANOVA) single factor, Games-Howell) (**B**). Plasma concentrations of IL-1RA in CFZ treated mice compared to untreated mice (*n* = 5 (CFZ groups); *n* = 4 (untreated group)) (* *p* < 0.05, analysis of variance (ANOVA) single factor, Games-Howell) (**C**).

In regards to the major side effect of oral CFZ therapy, the impact of the injected microparticles on skin pigmentation was quantitatively determined using a spectral reflectance assay as previously established [19]. Based on the skin reflectance signal acquired at the 480 nm and 623 nm optical filters, IV CFZ-HCl injected mice did not result in detected skin pigmentation (480 nm, no significant difference versus untreated mice, analysis of variance single factor, *n* = 4). Both untreated and IV injected mice exhibited significantly lower pigmentation than 9 week orally fed CFZ mice, with the 480 nm wavelength filter (*p* < 0.05, analysis of variance single factor, Games-Howell, *n* = 4) (Figure 7A,B). Importantly, the skin pigmentation of orally-fed mice is maximal at 3 weeks and then declines with continued feeding [19]; therefore, we can infer that IV injected mice are also less pigmented than mice fed with CFZ for 3 weeks. Additionally, the 623 nm optical filter (indicative of the presence of CFZ-HCl) was also unable to detect any significant skin pigmentation that may have arisen from partitioning of circulating CFZ-HCl particles from blood to skin [19]. Of noteworthy significance, the lack of skin pigmentation observed in the CFZ-HCl injected mice cannot be explained by an overt toxicological effect of the drug leading to decreased mice viability, as all mice survived the injection without obvious toxicological consequences. As a caveat, it is important to note that the extent of skin pigmentation is not necessarily a direct reflection of CFZ accumulation in the skin, since the spectral reflectance properties of the skin can also be impacted by the amount of CFZ that circulates through the skin capillaries, as well as the optical (absorbance) properties of the CFZ molecules which can change depending on the molecules’ ionization, aggregation, and redox states. Ultimately, the advantages of parenteral versus oral CFZ remain to be conclusively established under conditions leading to therapeutic efficacy, which will depend on the treatment regimen and clinical indication.

To further probe for the presence of toxic side effects resulting from the injected microparticles, TNFα, IL-1RA, and interleukin-1 beta (IL-1β) were measured. TNFα and IL-1β are pro-inflammatory cytokines that are released by cells, primarily macrophages, in response to inflammatory stimuli. IL-1RA is an early-acting acute-phase anti-inflammatory cytokine that dampens a broad spectrum of inflammatory conditions by inhibiting the activity of IL-1β, an important mediator of the inflammatory response [3,20]. However, none of these cytokines were significantly altered by an IV injection of CFZ-HCl (Figure 7C). In fact, IL-1RA levels were similar between treated and untreated groups (*p* < 0.05, analysis of variance single factor, Games-Howell, *n* = 5 (both CFZ groups), *n* = 4 (Control group); Figure 7C), while the plasma IL-1β and TNFα concentrations were below the limit of detection (IL-1β LOD = 12.5 pg/mL; TNFα LOD = 10.9 pg/mL).

## 4. Conclusions

In conclusion, a biocompatible, biomimetic formulation of CFZ-HCl microcrystals proved suitable for parenteral, systemic administration, while avoiding the skin pigmentation and immunological response that accompanies oral CFZ administration. Based on our in vitro and in vivo data, micronized CFZ-HCl crystals were ingested by tissue macrophages and accumulated in liver and spleen, similar to CLDIs; yet, no skin pigmentation was evident, unlike oral administration of CFZ. IV administration did not result in overt toxicity and did not induce measurable changes in acute inflammatory response biomarkers. The anti-inflammatory cytokine, IL-1RA, which we have previously shown to be elevated following oral administration of CFZ [3,20], was not changed by the injected CFZ-HCl microparticles. Our findings support additional studies into the possible use of parenteral CFZ-HCl as a means to circumvent the skin pigmentation side effect of the oral drug. For example, an inhalable, aerosolized formulation of CFZ-HCl may be a good strategy for treating MDR-TB. Previously, Brunaugh et al. [27] and Verma et al. [28] investigated the feasibility of inhalable micronized CFZ free base microparticles against MDR-TB. Nevertheless, based on a more straightforward synthesis route, scalable manufacturing, and other practical considerations, an inhalable formulation of CFZ-HCl microcrystals could offer a more viable approach for pharmaceutical product development. As a wise man once said: “the most fruitful basis for the discovery of a new drug is to start with an old drug [29,30,31]”.

## 5. Patents

Design and Composition of Cell-Stabilized Pharmaceutical Formulations. G.R. Rosania, P.J.A. Kenis, E.M. Horstman, T. Woldemichael, M.D. Murashov, P. Rzeczycki, R.K. Keswani, T.A. Arenson. 3 May 2018. Pub. No. WO/2018/081072. International Application No. PCT/US2017/058017

## Figures and Tables

**Table 1:** Quality and stability of single CFZ-HCl and CFZ free base micronized drug crystals measured by single crystal Raman micro-spectroscopy.

Sample (*n* = 3)	# Drug Crystals Scanned	# CFZ-HCl Crystals	# CFZ Free Base Crystals	# Other Particles
Micronized CFZ-HCl	100	98 ± 1	0	2 ± 1
Micronized CFZ-HCl in Diluent *	100	98 ± 1	0	2 ± 1
Micronized CFZ Free Base	100	0	97 ± 1	3 ± 1

* Diluent in IV injectable formulation for in vivo studies.
